# Progressive multifocal leukoencephalopathy reports in rheumatoid arthritis concerning different treatment patterns-an exploratory assessment using the food and drug administration adverse event reporting system

**DOI:** 10.3389/fdsfr.2024.1334468

**Published:** 2024-05-13

**Authors:** Takeshi Honma, Kenji Onda, Koichi Masuyama

**Affiliations:** ^1^ Bohsei Pharmacy, Kanagawa, Japan; ^2^ Department of Clinical Pharmacology, School of Pharmacy, Tokyo University of Pharmacy and Life Sciences, Tokyo, Japan; ^3^ Regulatory Science laboratory, School of Pharmacy, Tokyo University of Pharmacy and Life Sciences, Tokyo, Japan

**Keywords:** methotrexate, progressive multifocal leukoencephalopathy, rituximab, FDA adverse event reporting system, rheumatoid arthritis

## Abstract

**Introduction:** Progressive multifocal leukoencephalopathy (PML) is a rare but potentially life-threatening brain infection caused by the John Cunningham virus. PML is a known adverse effect associated with molecular-targeted drugs and immunosuppressive agents. Recent concerns have emerged regarding the link between methotrexate (MTX) and PML. However, limited information exists on the influence of concomitant drug use in rheumatoid arthritis (RA) treatment, where various medications are often used together.

**Methods:** To explore treatment patterns and patient background that affect PML reporting in RA, we analyzed data on RA cases from the Food and Drug Administration Adverse Event Reporting System (FAERS; JAPIC AERS) database between 1997 and 2019.

**Results and Discussion:** Our analysis revealed significantly elevated crude and adjusted reporting odds ratios (aROR) for MTX, rituximab (RIT), azathioprine, and cyclophosphamide. When considering treatment patterns, the concomitant use of MTX and RIT showed a higher aROR than using MTX or RIT alone. Additional TNF-α inhibitors or glucocorticoids did not increase PML reports. Moreover, male sex and older age were associated with increased PML reports. While limitations are inherent in studies using spontaneous reporting data, our exploratory assessment suggests an association between PML and the combination of MTX and RIT and a higher risk in men and older patients. These findings help enhance our understanding of PML risk factors in the context of RA treatment.

## 1 Introduction

Methotrexate (MTX) has been used as a cornerstone drug in the treatment of rheumatoid arthritis (RA). While MTX is highly effective against RA, its use necessitates consideration of potential side effects such as interstitial lung disease, hepatotoxicity, myelosuppression, and infections. The Pharmacovigilance Risk Assessment Committee of the European Medicines Agency (EMA) recently detected a signal indicating a potential association between MTX use and the development of progressive multifocal leukoencephalopathy (PML) (EMA, 2021). As a result, warnings regarding PML risk have been added to the labeling of MTX products in Europe and Japan.

PML is a severe, often fatal, infectious disease caused by the John Cunningham virus (JCV), typically due to declined cellular immunity (Cortese et al., 2021). Anti-JCV antibodies are present in over 80% of adults, with antibody prevalence increasing with age. PML occurs when latent JCV is reactivated, crosses the blood-brain barrier, enters the central nervous system, infects oligodendrocytes, and leads to JCV proliferation. This results in central nervous system damage characterized by cognitive impairment, speech difficulties, motor dysfunction, visual impairment, and other symptoms (Yukitake, 2018). PML has been primarily recognized as an opportunistic infection in patients with acquired immunodeficiency syndrome, with approximately 85% of cases in Europe and the United States having human immunodeficiency virus (HIV) infection as an underlying condition (Amend et al., 2010). However, the introduction of antiretroviral therapy has led to a reduction in HIV-related PML cases. Infection with JCV typically occurs during childhood, transmitted from parent to child, and increases with age (Cortese et al., 2021). In the general population, JCV seroprevalence reaches 60%–80% by the age of 70, with 50%–70% of individuals infected by adulthood. The presence of anti-JCV antibodies exceeds 80% in adults, with antibody prevalence increasing with age. The prevalence of PML in the general population is 0.2 in 100,000 (D’Amico et al., 2016). However, in patients with chronic inflammatory or autoimmune diseases, the risk of PML increases even in the absence of immunosuppressive therapies. The primary systemic diseases associated with PML are vasculitides, with the most prominent being Wegener’s granulomatosis and poly- or dermatomyositis, which increase to 2 in 100,000, and systemic lupus erythematosus, which increases to 4 in 100,000 (D’Amico et al., 2016).

In recent years, there has been an increase in drug-induced PML cases, occurring in various conditions such as hematologic disorders, malignancies, connective tissue diseases, autoimmune disorders such as multiple sclerosis, and posttransplant settings, often associated with the use of novel anticancer drugs, targeted therapies, and immunosuppressive agents (Cortese et al., 2021). Non-HIV drug-induced PML has been associated with various medications, including natalizumab, ephalizumab, fingolimod, and dimethyl fumarate, commonly used in multiple sclerosis treatment. However, there have also been reports of PML associated with drugs utilized in RA management, such as MTX, rituximab (RIT), oral glucocorticoid (GC), and tumor necrosis factor-α inhibitor (TNFi) (Berger et al., 2018).

Low-dose MTX for RA treatment and high doses for cancer therapy rarely lead to PML development. The precise mechanism of MTX affecting the brain remains unclear, but a hypothesis suggests that cumulative toxicity impairs the blood-brain barrier (Kougkas et al., 2022). Inhibition of tetrahydrofolate synthesis affecting the production of high-molecular-weight compounds like myelin, inhibition of dihydrofolate reductase, and reduced levels of S-adenosylmethionine are postulated as contributing factors to PML onset, with numerous complex interactions presumed to be involved.

RA treatment often involves a combination of drugs. MTX is a central component alongside biologic agents such as TNFis and disease-modifying antirheumatic drugs. Using these medications together, which impact immunity in different pathways, may potentially influence PML risk. However, there is a lack of information regarding how PML incidence varies with different treatment regimens, including those incorporating MTX. Such information is crucial for ensuring safe RA treatment.

The Food and Drug Administration (FDA) Adverse Event Reporting System (FAERS) is one of the largest spontaneous reporting adverse event database in the world, managed by the FDA. It collects information on adverse events associated with various pharmaceuticals (Sakaeda et al., 2013). Its utilization has been proposed for conducting disproportionality analysis for signal detection of drug adverse events and drug-drug interaction analysis (Bate and Evans, 2009; Gandhi et al., 2013; Zhao et al., 2013; Iyer et al., 2014; Nagaoka et al., 2021).

Due to the nature of spontaneous reporting, limitations such as reporting bias and the inability to aggregate the actual frequency of adverse effects exist. Therefore, it is inappropriate to compare the values of crude reporting odds ratios (cROR) obtained in disproportionality analysis. However, a methodology has been proposed involving multivariate analysis using logistic regression to calculate adjusted ROR (aROR) under specific conditions, enabling the comparison of reporting rates for adverse events (van Puijenbroek et al., 2000; Oshima et al., 2018; Onda et al., 2023).

In this study, we analyzed PML using FAERS from the following perspectives. First, we extracted RA cases from all reported cases in FAERS and aggregated cROR for all drugs related to PML through disproportionality analysis. Next, to examine the comedications influencing PML reporting, we used age, sex, and RA treatment drugs with high cROR values as explanatory variables. We calculated aROR for PML using multiple logistic regression analysis (model 1). Furthermore, to facilitate comparisons across various RA treatment patterns, we performed multivariate analyses (model 2, model 3) in patients with RA. These analyses considered age, sex, and the treatment patterns of MTX and other comedications (RIT, azathioprine [AZA], cyclophosphamide [CPA], GCs, and TNFis) as explanatory variables. Based on these analyses, we evaluated whether differences in treatment regimens, including MTX and its comedications, had an impact on PML reporting in patients with RA.

## 2 Materials and methods

### 2.1 Data source and mining

We conducted an analysis using the Japan Pharmaceutical Information Center (JAPIC) AERS based on data from the FDA Adverse Event Reporting System (FAERS). The data span from the fourth quarter of 1997 to the first quarter of 2019, with duplicate reports removed. Since FAERS is an anonymized public database, approval from an institutional review board was exempted. We utilized four tables from the FAERS database, namely, DEMO (demographic information), DRUG (medications), REAC (adverse events), and INDI (indications). These tables were interconnected using the PrimaryID and analyzed using relational database software (Microsoft Access 2016).

### 2.2 Definition of adverse events

All adverse events were extracted using Preferred Terms (PT) in the Medical Dictionary for Regulatory Activities (MedDRA) version 22.0. PML was defined using a single PT code, 10036807.

### 2.3 Disproportionality analysis

**FIGURE 1:**
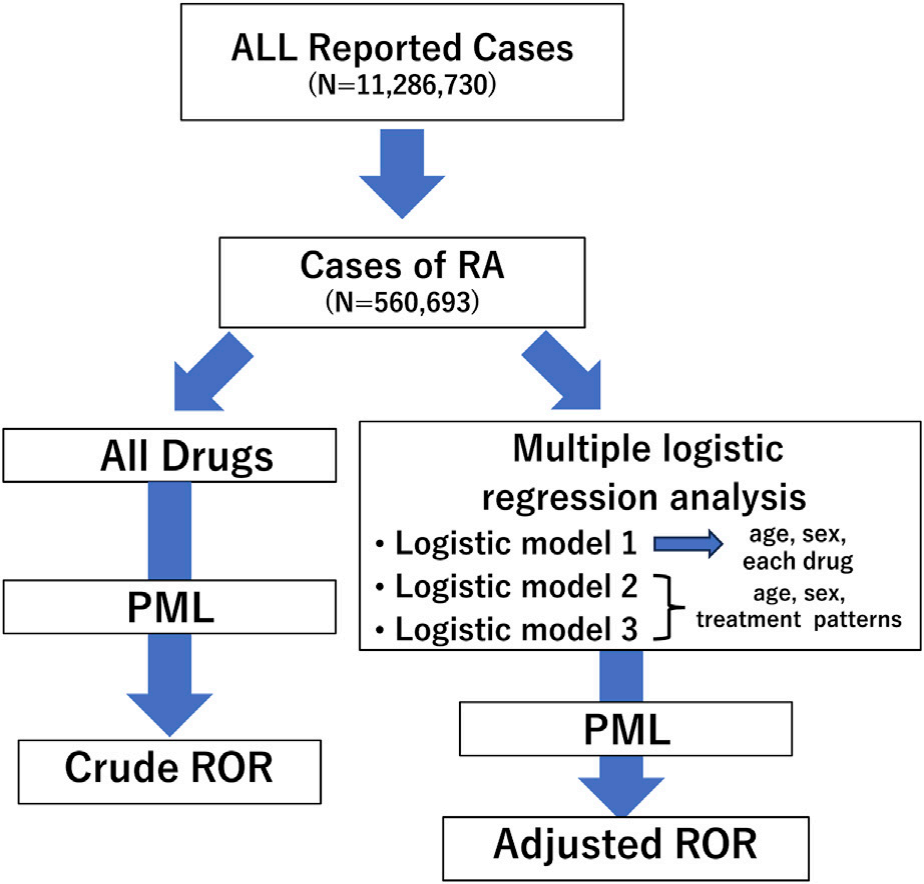
Flow chart for determining crude reporting odds ratios (cRORs) and adjusted RORs (aRORs) of events on progressive multifocal leukoencephalopathy (PML) in the study. A disproportionality analysis was performed focusing on PML events considering all rheumatoid arthritis cases. Next, we selected patients aged ≤100 years and conducted multiple logistic regression analyses to calculate aRORs for the adverse event. We set PML adverse event as the objective variable and age, sex, and treatment patterns as explanatory variables. RORs, reporting odds ratios; PML, progressive multifocal leukoencephalopathy; RA, rheumatoid arthritis.

**FIGURE 2:**
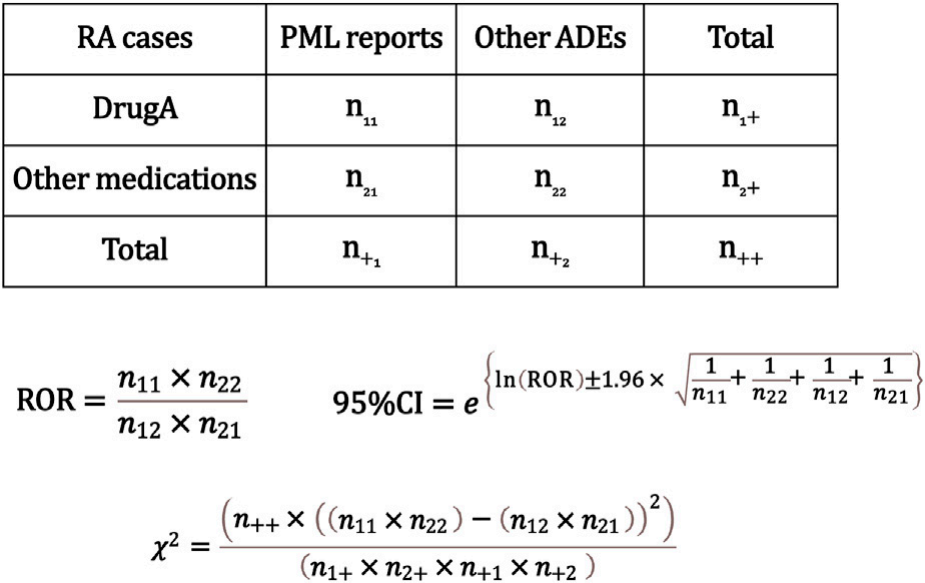
Calculation of the crude reporting odds ratios (cRORs), 95% confidence intervals, and χ2 values for progressive multifocal leukoencephalopathy (PML) event based on 2 × 2 contingency tables. The cRORs for PML events were calculated with the 2 × 2 contingency table among cases of rheumatoid arthritis. RA, rheumatoid arthritis; PML, progressive multifocal leukoencephalopathy; ADE, adverse drug event; ROR, reporting odds ratio; CI, confidence interval.

The analysis was conducted following the flowchart presented in Figure 1. Initially, we extracted RA cases from all reported cases using the INDI table and performed disproportionality analysis focusing on PML reports. We created a 2 × 2 contingency table from the total number of RA cases in which each registered drug was used and PML was reported. Subsequently, we calculated the cROR, 95% confidence intervals (CI), and χ^2^ value (Figure 2).

### 2.4 Multiple logistic regression analysis of progressive multifocal leukoencephalopathy reports for each drug in patients with rheumatoid arthritis

Next, we conducted a multiple logistic regression analysis to investigate the aRORs of various drugs with respect to the number of PML reports, excluding cases with unknown sex and limiting the dataset to individuals aged ≤100 years. This analysis was performed using data from the DEMO and INDI tables. The presence or absence of PML reports served as the objective variable. We included age, sex, and high cROR drugs identified through the disproportionality analysis, which included MTX, RIT, AZA, CPA, leflunomide, hydroxychloroquine, infliximab, prednisone, prednisolone, and methylprednisolone as explanatory variables. Additionally, we included TNFis with high reporting numbers, such as etanercept, adalimumab, golimumab, and certolizumab pegol, as explanatory variables in our logistic model Eq. 1.

$$\mathrm{Log}\;\left(RORs\right)=\beta_{0}+\beta_{1}A+\beta_{2}S+\beta_{3}D_{1}+\cdots +\beta_{12}D_{10}$$ (1)

(A = age, S = sex, D_1_–D_10_ = drugs).

### 2.5 Multiple logistic regression analysis of progressive multifocal leukoencephalopathy reports with different treatment patterns in patients with rheumatoid arthritis

Next, we analyzed the impact of treatment patterns using the drugs on PML reports. The analysis of different drug treatment patterns was based on the model equation defined in our previous report (Onda et al., 2023), rooted in the definition of combination patterns. When analyzing combinations of multiple drugs, the explanatory variables for the logistic model can become complex. Therefore, in this analysis, we divided it into two cases and conducted the analysis using respective model Eq. 2, Eq. 3.

First, we focused on four drugs, namely, MTX, RIT, AZA, and CPA, which exhibited particularly significant aROR values in the previous analysis (Table 2). Depending on treatment patterns, RA cases were categorized into seven groups: 1) MTX group (no combination with RIT, CPA, or AZA), 2) RIT group (no combination with MTX, CPA, or AZA), 3) AZA group (no combination with MTX, RIT, or CPA), 4) CPA group (no combination with MTX, RIT, or AZA), 5) MTX + RIT combination group, 6) MTX + AZA combination group, and 7) MTX + CPA combination group. The four drug combination patterns were defined, and explanatory variables were set based on the presence of these combination patterns. We then performed a multiple logistic regression analysis using the following model Eq. 2.

$$\mathrm{Log}\;\left(RORs\right)=\beta_{0}+\beta_{1}A+\beta_{2}S+\beta_{3}T_{1}\cdots +\beta_{9}T_{7}$$ (2)

(A = age, S = sex, T_1_ = MTX (no RIT, no CPA, no AZA), T_2_ = RIT (no MTX, no CPA, no AZA), T_3_ = AZA (no MTX, no RIT no CPA), T_4_ = CPA (no MTX, no RIT, no AZA), T_5_ = MTX + RIT (no AZA, no CPA), T_6_ = MTX + AZA (no RIT, no CPA), T_7_ = MTX + CPA (no RIT, no AZA).

Furthermore, we focused on the drugs with many reports, namely, MTX, RIT, GC, and TNFis in Table 2. We performed a multiple logistic regression analysis to investigate the effect of these four drug combinations. The explanatory variables in this analysis were as follows: 1) MTX group (no RIT, GC, or TNFi), 2) RIT group (no MTX, GC, or TNFi), 3) MTX + RIT combination group (no GC or TNFi), 4) MTX + RIT + GC combination group (no TNFi), 5) MTX + RIT + TNFi combination group (no GC), and 6) MTX + RIT + GC + TNFi combination group. We defined the RA cases within each group. Explanatory variables were set based on the presence of these combination patterns, and a multiple logistic regression analysis was conducted using the following model Eq. 3. TNFi usage was defined as using one of the following: infliximab, etanercept, adalimumab, golimumab, or certolizumab pegol. GC usage was defined as using one of the following: prednisolone, methylprednisolone, or prednisone.

$$\mathrm{Log}\;\left(RORs\right)=\beta_{0}+\beta_{1}A+\beta_{2}S+\beta_{3}T_{1}\cdots +\beta_{8}T_{6}$$ (3)

(A = age, S = sex, T_1_ = MTX (no RIT, GC, TNFi), T_2_ = RIT (no MTX, GC, TNFi), T_3_ = MTX + RIT (no GC, TNFi), T_4_ = MTX + RIT + GC (no TNFi) T_5_ = MTX + RIT + TNFi (no TNFi), T_6_ = MTX + RIT + TNFi + GC).

Statistical significance was determined when the upper 95% CI of ROR was <1.0 or when the lower 95% CI of ROR was >1.0. Data mining and all statistical analyses were conducted using Microsoft Access 2016, R version 3.4.1, and EZR version 1.36 (Kanda, 2013).

## 3 Results

### 3.1 Crude reported odds ratio of progressive multifocal leukoencephalopathy in rheumatoid arthritis cases

The total number of reported cases in the cleaned FAERS dataset was 11,286,730. Among these, 560,693 were RA cases. There were 186 PML reports among the RA cases. A disproportionality analysis was conducted between the use of all registered drugs and PML reports for these cases, and the representative results are presented in Table 1. Among drugs with more than 10 PML reports, the cROR (95% CI) was significantly high for the following drugs: MTX (3.72 [2.78–4.99]), RIT (35.42 [26.55–47.27]), prednisone (2.35 [1.65–3.33]), infliximab (3.16 [2.19–4.56]), hydroxychloroquine (2.90 [2.0–4.2]), prednisolone (4.82 [3.26–7.12]), leflunomide (2.73 [1.84–4.03]), AZA (14.41 [9.06–22.94]), methylprednisolone (7.55 [4.64–12.29]), and CPA (70.26 [37.97–130.02]).

**TABLE 1:** Crude reporting odds ratio (cROR) of PML in patients with RA.

Drug	Cases	Non-cases		Cases	Non-cases		Crude ROR (95% CI)	Χ^2^
	n_11_	n_12_	%	n_21_	n_22_	%		
Methotrexate	109	154,381	0.07	77	406,126	0.02	3.72 (2.78–4.99)	89.86
Rituximab	99	17,445	0.57	87	543,062	0.02	35.42 (26.55–47.27)	1540.57
Prednisone	40	58,566	0.07	146	501,941	0.03	2.35 (1.65–3.33)	24.29
Infliximab	35	38,321	0.09	151	522,186	0.03	3.16 (2.19–4.56)	41.88
Hydroxychloroquine	34	40,162	0.08	152	520,345	0.03	2.9 (2.0–4.2)	34.51
Prednisolone	30	21,522	0.14	156	538,985	0.03	4.82 (3.26–7.12)	75.98
Leflunomide	30	36,926	0.08	156	523,581	0.03	2.73 (1.84–4.03)	27.49
Etanercept	24	255,228	0.01	162	305,279	0.05	0.18 (0.12–0.27)	79.84
Folic acid	24	57,252	0.04	162	503,255	0.03	1.3 (0.85–2.0)	1.47
Azathioprine	20	4,646	0.43	166	555,861	0.03	14.41 (9.06–22.94)	221.89
Adalimumab	20	149,341	0.01	166	411,166	0.04	0.33 (0.21–0.53)	24.03
Methylprednisolone	18	7,841	0.23	168	552,666	0.03	7.55 (4.64–12.29)	92.21
Acetylsalicylic acid	14	19,813	0.07	172	540,694	0.03	2.22 (1.29–3.83)	8.69
Paracetamol	13	33,219	0.04	173	527,288	0.03	1.19 (0.68–2.1)	0.38
Cyclophosphamide	11	501	2.20	175	560,006	0.03	70.26 (37.97–130.02)	691.44
Omeprazole	11	18,974	0.06	175	541,533	0.03	1.79 (0.98–3.3)	3.63
Tocilizumab	11	27,465	0.04	175	533,042	0.03	1.22 (0.66–2.24)	0.41
Alendronate sodium	10	10,384	0.10	176	550,123	0.03	3.01 (1.59–5.69)	12.69
Colecalciferol	10	13,761	0.07	176	546,746	0.03	2.26 (1.19–4.27)	6.62
Levothyroxine	10	22,381	0.04	176	538,126	0.03	1.37 (0.72–2.58)	0.93

ROR, reporting odds ratio; PML, progressive multifocal leukoencephalopathy; RA, rheumatoid arthritis.

### 3.2 Multiple logistic regression analysis of progressive multifocal leukoencephalopathy reports for each drug in patients with rheumatoid arthritis

Subsequently, a multiple logistic regression was performed on a subset of 379,021 RA cases with patients aged ≤100 years and known sex. There were 153 PML cases selected. This analysis assessed the effect of drugs with significantly high cROR values and TNFis usage, which had many reports on PML reporting, as detailed in Table 2.

**TABLE 2:** Multiple logistic regression analysis (logistic model 1) to determine adjusted reporting odds ratio (aROR) for PML in patients with RA.

Variables		Non-cases	Cases	Proportion (%)	Adjusted ROR	*p*-value
Age, mean (SD)		58.25 (13.42)	64.82 (11.09)		1.03 (1.02–1.05)	<0.001
Sex	Male	73,680	49	0.07 (0.05–0.09)	1.61 (1.13–2.29)	0.008
	Female	305,341	104	0.03 (0.03–0.04)		
Methotrexate		104,175	95	0.09 (0.07–0.11)	2.39 (1.7–3.37)	<0.001
Rituximab		10,469	79	0.75 (0.59–0.93)	18.4 (12.8–26.4)	<0.001
Azathioprine		2,998	20	0.66 (0.41–1.02)	5.5 (3.23–9.38)	<0.001
Cyclophosphamide		370	10	2.63 (1.27–4.79)	4.6 (2.18–9.7)	<0.001
Prednisone		42,470	34	0.08 (0.06–0.11)	1.06 (0.71–1.59)	0.772
Prednisolone		16,225	26	0.16 (0.1–0.23)	1.45 (0.90–2.33)	0.125
Methylprednisolone		6,193	16	0.26 (0.15–0.42)	0.93 (0.53–1.63)	0.805
Hydroxychloroquine		26,225	32	0.12 (0.08–0.17)	1.38 (0.87–2.17)	0.172
Leflunomide		24,411	26	0.11 (0.07–0.16)	1.22 (0.75–2.0)	0.424
Infliximab		22,456	28	0.13 (0.08–0.18)	1.68 (1.07–2.62)	0.023
Etanercept		181,935	20	0.01 (0.01–0.02)	0.20 (0.12–0.34)	<0.001
Adalimumab		88,747	17	0.02 (0.01–0.03)	0.40 (0.24–0.67)	<0.001
Golimumab		10,109	4	0.04 (0.01–0.1)	0.76 (0.28–2.08)	0.591
Certolizumab pegol		11,138	1	0.01 (0–0.05)	0.14 (0.02–1.04)	0.055

ROR, reporting odds ratio; PML, progressive multifocal leukoencephalopathy; RA, rheumatoid arthritis.

The results showed that higher age, with an aROR (95% CI) of 1.03 (1.02–1.05), and male sex, with an aROR of 1.61 (1.13–2.29), were both significantly associated with high PML reporting.

Additionally, the analysis revealed that several drugs had significantly high aROR (95% CI) values, indicating a greater association with PML reports than the others. These included MTX (2.39 [1.70–3.37]), RIT (18.4 [12.8–26.4]), CPA (5.5 [3.23–9.38]). Infliximab (1.68 [1.07–2.62]) showed a statistically significant association with PML reporting. However, other drugs, such as prednisone (1.06 [0.71–1.59]), prednisolone (1.45 [0.90–2.33]), methylprednisolone (0.93 [0.53–1.63]), hydroxychloroquine (1.38 [0.87–2.17]), leflunomide (1.22 [0.75–2.00]), etanercept (0.20 [0.12–0.34]), adalimumab (0.40 [0.24–0.67]), golimumab (0.76 [0.28–2.08]), and certolizumab pegol (0.14 [0.02–1.04]), were not associated with high PML reporting.

### 3.3 Multiple logistic regression analysis of progressive multifocal leukoencephalopathy reports for treatment patterns containing methotrexate, rituximab, azathioprine, and cyclophosphamide in patients with rheumatoid arthritis

Subsequently, we focused on MTX, RIT, AZA, and CPA, which exhibited significantly high aROR values, to examine the impact of their combinations on PML reporting. Each case was categorized based on the treatment patterns defined in the materials and method section, and a multiple logistic regression analysis was conducted using model 2.

The results revealed that the aRORs (95% CI) for the MTX and RIT groups were 3.32 (2.06–5.37) and 43.4 (24.8–76.0), respectively. The aRORs for the AZA and CPA groups were 49.0 (23.9–101.0) and 233.0 (69.5–781.0), respectively. The MTX + RIT group had an aROR of 76.9 (48.8–121.0), higher than that of the MTX and RIT groups (Table 3).

**TABLE 3:** Multiple logistic regression analysis (logistic model 2) of PML reports for treatment patterns containing methotrexate, rituximab, cyclophosphamide, and azathioprine in RA patients.

Variables		Non-cases	Cases	Proportion (%)	Adjusted ROR	*p*-value
Age, mean (SD)		58.25 (13.42)	64.82 (11.09)		1.04 (1.03–1.05)	<0.001
Sex	Male	73,680	49	0.07 (0.05–0.09)	1.74 (1.24–2.45)	0.002
	Female	305,341	104	0.03 (0.03–0.04)		
MTX (no RIT, no CPA, no AZA)		97,176	38	0.04 (0.03–0.05)	3.32 (2.06–5.37)	<0.001
RIT (no MTX, no CPA, no AZA)		3,983	21	0.52 (0.33–0.8)	43.4 (24.8–76.0)	<0.001
AZA (no MTX, no RIT no CPA)		1,718	10	0.58 (0.28–1.06)	49.0 (23.9–101.0)	<0.001
CPA (no MTX, no RIT, no AZA)		97	3	3 (0.62–8.52)	233.0 (69.5–781.0)	<0.001
MTX + RIT (no AZA, no CPA)		5,749	49	0.85 (0.63–1.12)	76.9 (48.8–121.0)	<0.001
MTX + AZA (no RIT, no CPA)		636	1	0.16 (0–0.87)	15.3 (2.09–113.0)	0.007
MTX + CPA (no RIT, no AZA)		94	1	1.05 (0.03–5.73)	90.2 (12.1–671.0)	<0.001

ROR, reporting odds ratio; PML, progressive multifocal leukoencephalopathy; RA, rheumatoid arthritis; MTX, methotrexate; RIT, rituximab; AZA, azathioprine; CPA, cyclophosphamide.

The aRORs for the MTX + AZA and MTX + CPA groups were 15.3 (2.09–113.0) and 90.2 (12.1–671.0), respectively. These values were higher compared with those of the MTX group. However, the number of PML reports in these combination groups was only one case each, lower than that of the AZA and CPA groups.

### 3.4 Multiple logistic regression analysis of progressive multifocal leukoencephalopathy reports for treatment patterns containing methotrexate, rituximab, glucocorticoids, and tumor necrosis factor-α inhibitors in patients with rheumatoid arthritis

Considering the observed increase in aROR when combining MTX and RIT, we explored the potential effects of other combination drugs with a significant number of reports. We utilized explanatory variables that categorized treatment patterns based on MTX, RIT, GCs, and TNFis usage and conducted a multiple logistic regression analysis.

The results of the analysis using model 3 revealed the following aROR (95% CI) values: MTX group, 1.14 (0.50–2.62) and RIT group, 24.6 (13.4–45.2). The MTX + RIT group displayed the highest aROR at 97.4 (63.0–151.0). Furthermore, the aRORs for the MTX + RIT + GC, MTX + RIT + TNFi, and MTX + RIT + GC + TNFi groups were 30.3 (16.1–57.2), 25.9 (14.1–47.6), and 14.1 (5.17–38.7), respectively (Table 4).

**TABLE 4:** Multiple logistic regression analysis (logistic model 3) of PML reports for treatment patterns containing methotrexate, rituximab, glucocorticoids and TNF-α inhibitors in patients with RA.

Variables		Non-cases	Cases	Proportion (%)	Adjusted ROR	*p*-value
Age, mean (SD)		58.25 (13.42)	64.82 (11.09)		1.04 (1.02–1.05)	<0.001
Sex	Male	73,680	49	0.07 (0.05–0.09)	1.85 (1.32–2.61)	<0.001
	Female	305,341	104	0.03 (0.03–0.04)		
MTX (no RIT, no GC, no TNFi)		20,349	6	0.03 (0.01–0.06)	1.14 (0.50–2.62)	0.755
RIT (no MTX, no GC, no TNFi)		2,029	12	0.59 (0.3–1.03)	24.6 (13.4–45.2)	<0.001
MTX + RIT (no GC, no TNFi)		1,149	28	2.43 (1.59–3.42)	97.4 (63.0–151.0)	<0.001
MTX + RIT + GC (no TNFi)		1,506	11	0.73 (0.36–1.29)	30.3 (16.1–57.2)	<0.001
MTX + RIT + TNFi (no GC)		2,218	12	0.54 (0.28–0.94)	25.9 (14.1–47.6)	<0.001
MTX + RIT + GC + TNFi		1364	4	0.29 (0.08–0.75)	14.1 (5.17–38.7)	<0.001

ROR, reporting odds ratio; PML, progressive multifocal leukoencephalopathy; RA, rheumatoid arthritis; MTX, methotrexate; RIT, rituximab; GC, glucocorticoid; TNFi, tumor necrosis factor-α inhibitor.

## 4 Discussion

Drug-induced PML has been reported to have a 70%–80% survival rate when early detection and immune reconstitution are pursued (Clifford et al., 2011). Therefore, risk information for each specific drug and insights into the impact of drug combinations are of paramount importance.

Previous reports of drug-induced PML using the FAERS database have focused on all kinds of diseases, multiple sclerosis, or multiple myeloma; however, none have centered on RA (Raisch et al., 2016; Focosi et al., 2019; Oshima et al., 2019; Jonasson et al., 2023). This study analyzed 153 cases of PML reports from the perspective of both individual drugs and drug combinations in RA cases using the large-scale spontaneous reporting database, FAERS.

Initially, we conducted a comprehensive disproportionality analysis of individual drugs (univariate analysis), calculating the cROR for PML for all medication in RA cases, which allowed us to confirm signal detection. Subsequently, through multivariate analysis, we used the logistic model 1 with the presence or absence of each drug as explanatory variables to calculate the aROR for each drug. Furthermore, using drug combination patterns based on these medications as explanatory variables, we calculated the aROR for PML. We analyzed the impact of drug combinations on adverse event reporting (with logistic models 2 and 3). Our previous analysis, based on information registered in FAERS, confirmed that the maximum MTX weekly dose in RA cases is predominantly distributed below 25 mg (Onda et al., 2023). Therefore, this analysis focused on cases of low-dose MTX use.

Various methods have been proposed to detect adverse event signals during drug combination analysis in spontaneous reporting databases. While the Ω shrinkage measure model and other approaches are designed to identify drug-drug interactions between two drugs (Norén et al., 2008; Noguchi et al., 2019), our study focused on cases involving three or more drugs. Thus, we employed multiple logistic regression analysis (van Puijenbroek et al., 2000; Onda et al., 2023). In the multivariate analysis of drug treatment patterns, we used a model based on the approach we reported earlier and set explanatory variables for each treatment pattern (Onda et al., 2023). In the logistic models used in this study, all variance inflation factors were <1.8, confirming the absence of multicollinearity issues. The statistical deviance was significantly small (*p < 0.001*) in all logistic models. In the case of logistic analysis with low event rate, sparse data bias could be concerned (Gosho et al., 2023). We confirmed that all logistic analyses ruled out complete or quasi-complete separation. These information support the validity of the analysis.

Drugs that exhibited significantly elevated cROR values in the disproportionality analysis included MTX, RIT, prednisone, infliximab, hydroxychloroquine, prednisolone, leflunomide, AZA, methylprednisolone, and CPA. These findings suggest a potential association between drugs that impact the immune system used for patients with RA and the development of PML. However, cROR values calculated through disproportionality analysis may not account for confounding factors such as coadministered drugs, making direct comparisons inappropriate (Urushihara, 2020). Therefore, in the subsequent analyses, we conducted multiple logistic regression analyses (logistic models 1, 2, and 3) to assess the influence of drugs on PML reports in RA cases, focusing on drug usage and combinations.

After extracting RA cases with patients aged ≤100 years, we conducted a multiple logistic regression analysis with age, sex, and the presence or absence of each drug as explanatory variables. The results showed that men and older age were significantly associated with PML reports in all models. Within the context of a predominantly women-RA population, older men may be a noteworthy group of patients at risk for PML onset.

In the analysis of logistic model 1, which included explanatory variables for each drug, the aROR for MTX usage was significantly elevated, implying an association between low-dose MTX, commonly used in RA and PML reports. Additionally, RIT, CPA, and AZA exhibited high aRORs, indicating the association of PML reports with these drugs.

Afterward, using logistic model 2, we analyzed the effect of drug combination patterns on PML reports in RA cases. In this model, we primarily examined the effects of combining MTX with RIT, AZA, or CPA (Table 3). The results showed that compared with the MTX group, the aROR for PML reports were higher in the MTX + RIT, MTX + AZA, and MTX + CPA groups, suggesting an increased risk of PML when combining MTX with these drugs.

We then analyzed the impact of additional concomitant use of GCs and TNFis using logistic model 3. In the logistic model 2, the MTX + RIT group had a higher aROR than the MTX group, and the PML case count in the MTX + RIT group was higher than that in the MTX + CPA and MTX + AZA groups. Therefore, we focused on the MTX + RIT group and examined the effects of adding other concomitant drugs. We conducted a multiple logistic regression analysis by including GCs and TNFis, which had high cROR values, as explanatory variables.

The results revealed that, compared with the MTX group, all groups in which RIT was used showed higher aROR values. Furthermore, when GCs, TNFis, or both were added to the MTX + RIT group, there was no increase in aROR compared to the aROR observed in the MTX + RIT group. Based on the results of logistic models 2 and 3, combining RIT with MTX increased PML reports compared with MTX monotherapy. However, adding TNFis, GCs, or both did not increase PML reports in the MTX + RIT group.

RIT, an anti-CD20 antibody, is used not only in treating refractory RA but also in various malignant tumors, including lymphomas. Previous analyses of PML using FAERS data have consistently shown an association between RIT usage and PML reports (Raisch et al., 2016; Oshima et al., 2019; Jonasson et al., 2023). PML incidence rate is estimated to be 1.39–1.87 per 10,000 people in patients with lymphoma and autoimmune disease when RIT is used (Focosi et al., 2019).

Clifford et al. reported four PML cases related to RIT use in patients with RA and estimated PML occurrence associated with RIT use in patients with RA to be 1 in 25,000 individuals (Clifford et al., 2011). However, the number of reported PML cases did not increase after the expansion of RIT use, suggesting the involvement of multiple independent factors unrelated to RIT treatment (Berger et al., 2018).

Regarding the association between MTX use in patients with RA and PML, Kougkas et al. recently reported a PML case in a patient with RA receiving combination therapy of MTX and RIT, along with four previous cases of MTX-related PML (Kougkas et al., 2022). Our study represents the first report to investigate treatment patterns associated with accumulated PML reports in RA cases using the large-scale FAERS data. As a result, we demonstrated a significant association between MTX or RIT usage and PML reports through multivariate analysis. Furthermore, our analysis of combination patterns revealed new findings, such as higher aROR for RIT compared with that of MTX and even higher aROR for PML when MTX and RIT were used in combination, as compared to their individual use. Older age, male sex, and the concomitant use of MTX and RIT suggest the necessity of monitoring and vigilance for PML detection.

Spontaneous reporting databases like FAERS inherently suffer from an unknown denominator, making it impossible to assess the actual frequency of adverse events. Moreover, it is crucial to acknowledge the various reporting biases. Therefore, aROR calculation and the observations made in this study may not necessarily reflect the actual situation. While this study employed multiple logistic regression models for analysis, it is important to note that aROR comparison should be performed within the same model, and a direct comparison of aROR between different model formulations is not valid.

Furthermore, this analysis did not account for factors such as dosage, the timing and route of administration, and disease severity. Additionally, it is not possible to eliminate the influence of potential confounding factors that were not included in the logistic regression model. Despite its limitations, FAERS remains a vital research tool for analyzing rare side effects like PML. Analysis focusing on a population in a large-scale data may reveal potential influence that are unnoticed in analyses of individual cases. Investigation of case series based on our finding enhances understanding of causal relationship between drug exposure and PML onset.

## 5 Conclusion

In the current study, we explored treatment patterns of patient with RA and patient demographic background (age, sex) that affect PML reporting. To the best of our knowledge, this is the first study to analyze the impact of RA pharmacotherapy on PML using FAERS data. We demonstrated associations between older age, male sex, and MTX use with PML reports in patients with RA. Furthermore, the concomitant use of MTX and RIT potentially increases PML risk compared with their individual use. This research provides valuable insights into the safe pharmacological management of RA.

## Data Availability

Publicly available datasets were analyzed in this study. This data can be found here: https://www.fda.gov/drugs/questions-and-answers-fdas-adverse-event-reporting-system-faers/fda-adverse-event-reporting-system-faers-public-dashboard.
